# Effectiveness of an attachment‐based intervention for the assessment of parenting capacities in maltreating families: A randomized controlled trial

**DOI:** 10.1002/imhj.21874

**Published:** 2020-06-24

**Authors:** Sabine van der Asdonk, Whitney D. de Haan, Sheila R. van Berkel, Marinus H. van IJzendoorn, Ralph C. A. Rippe, Carlo Schuengel, Chris Kuiper, Ramon J. L. Lindauer, Mathilde Overbeek, Lenneke R. A. Alink

**Affiliations:** ^1^ Institute of Education and Child Studies Leiden University Leiden The Netherlands; ^2^ Private Law Vrije Universiteit Amsterdam Amsterdam The Netherlands; ^3^ Department of Psychology, Education and Child Studies Erasmus University Rotterdam Rotterdam The Netherlands; ^4^ Primary Care Unit, School of Clinical Medicine University of Cambridge Cambridge United Kingdom; ^5^ Clinical Child and Family Studies Vrije Universiteit Amsterdam Amsterdam The Netherlands; ^6^ Youth Expert Center University of Applied Sciences Leiden The Netherlands; ^7^ Horizon Youth Care and Education Rotterdam The Netherlands; ^8^ Department of Child and Adolescent Psychiatry, Academic Medical Center University of Amsterdam Amsterdam The Netherlands; ^9^ De Bascule Academic Center for Child and Adolescent Psychiatry Amsterdam The Netherlands; ^10^ Yulius Mental Health Clinic Rotterdam The Netherlands

**Keywords:** attachment‐based intervention, child maltreatment, parenting capacity, placement decisions, RCT, maltrato infantil, capacidad de crianza, intervención con base en la afectividad, RCT, toma de decisión, Parent, bébé, neurodéveloppement, ajustement, intervention de psychologie, Kindesmisshandlung, elterliche Kapazitäten, bindungsbasierte Intervention, RCT, Entscheidungsfindung, 児童虐待, 育児能力, アタッチメントに基づく介入, RCT, 意思決定, 儿童虐待, 育儿能力, 依恋干预, 随机对照试验, 决策, تنظيم الإجهاد، وإيقاع الكورتيزول اليومي، والتعلق بمقدم الرعاية، والرعاية خارج المنزل

## Abstract

Even though Parenting Capacity Assessments (PCAs) are essential for child protection services to support placement decisions for maltreating families, presently no evidence‐based PCA protocols are available. In this randomized controlled trial, we tested the quality of an attachment‐based PCA protocol based on Video‐feedback Intervention to promote Positive Parenting and Sensitive Discipline (VIPP‐SD). We recruited 56 parent‐child dyads (*M*_age_ children = 3.48 years) in Dutch family residential clinics that conduct PCAs to support placement decisions. After pretest, families were randomized to receive the Regular Assessment Procedure (RAP) (*n* = 28), or an additional assessment based on VIPP‐SD (*n* = 28). An immediate post‐test and a 10‐month follow‐up were conducted. Multilevel models showed that therapists felt equally confident about their recommendations regarding child placement for both groups and that they equally often modified their initial placement recommendations. Moreover, children in the VIPP‐SD group did not show fewer behavior problems and did not experience recurring child maltreatment less often than children in the RAP group. Thus, we found no evidence that PCAs incorporating the VIPP‐SD protocol outperformed PCAs as usual. We discuss possible explanations why in the current study VIPP‐SD did not seem to add to the quality of the RAP.

## RESUMEN

A pesar de que las Evaluaciones de Capacidad de Crianza (PCA) son esenciales para los servicios de protección al niño para apoyar las decisiones de dónde colocarlo por razones de familias maltratadoras, en el presente no se encuentran disponibles ningún protocolo PCA basado en la evidencia. En este ensayo controlado al azar, pusimos a prueba la calidad de un protocolo PCA basado en la afectividad para lo cual nos basamos en la Intervención de Video Informativo para promover una Crianza Positiva y Disciplina Sensible (VIPP‐SD). Reclutamos 56 díadas de progenitor‐niño (edad promedio de los niños = 3.48 años) en clínicas residenciales de familias holandesas que utilizan las PCA para apoyar las decisiones de dónde colocar. Después del pre‐examen, las familias fueron asignadas al azar para recibir el Procedimiento de Evaluación Regular (RAP) (n = 28), o una evaluación adicional basada en VIPP‐SD (n = 28). Se llevaron a cabo un examen posterior inmediato y un seguimiento a los 10 meses. Los modelos de niveles múltiples mostraron que los terapeutas se sintieron igualmente confiados acerca de sus recomendaciones con respecto a dónde colocar al niño en ambos grupos y que ellos igualmente modificaron a menudo su recomendación inicial de dónde colocarlo. Es más, los niños en el grupo VIPP‐SD no mostraron menos problemas de conducta y no experimentaron maltrato infantil recurrente menos a menudo que los niños en el grupo RAP. Por tanto, no encontramos evidencia de que las PCA que incorporan el protocolo VIPP‐SD tuvieran mejores resultados que las PCA en su forma usual. Discutimos posibles explicaciones de por qué en el presente estudio los VIPP‐SD no parecieron agregar nada a la calidad de RAP.

## RÉSUMÉ

Contexte: Des interventions favorables et ciblées pour les familles sont nécessaires afin d'optimiser l'ajustement parental et la relation parent‐bébé conformément à un diagnostic précédant de risque neurodéveloppemental pour les bébés. Buts: Le but de cette revue systématique était de déterminer l'efficacité des interventions pour l'amélioration de l'ajustement psychologique et le bien‐être pour les parents ayant un bébé ayant été diagnostiqué comme ayant ou étant à risque d'avoir un trouble neurodéveloppemental. Méthodes: La stratégie de recherche du Cochrane Review Group a été suivie avec une recherche des essais contrôlés du Registre Cochrane Central, de PubMed, CINAHL, PsycINFO, et Embase entre juillet et décembre 2017. La qualité méthodologique des articles inclus a été évaluée au moyen de l’échelle de la base de données de preuve de physiothérapie (PEDro Physiotherapy Evidence Database) par deux évaluateurs indépendants. Résultats: Douze études ont rempli les critères d'inclusion. Un petit nombre d'essais de grande qualité ont révélé une efficacité modérée à importante de la réduction chez les parents de symptômes psychologiques indésirables de trauma et de stress des parents. Des améliorations importantes dans les symptômes de dépression et d'anxiété ont émergé lors de follow up post intervention à plus long terme (6 mois‐8 ans). Conclusions: On constate un soutien prometteur pour l'efficacité de certaines interventions à réduire les symptômes psychologiques de mésadaptation chez les parents avec des bébés diagnostiqués comme étant à risque d'un trouble neurodéveloppemental. D'autres ECR de qualité d'intervention psychologiques portant sur les conditions plus grandes de risque neurodéveloppemental sont nécessaires.

## ZUSAMMENFASSUNG

Die Wirksamkeit einer bindungsbasierten Intervention zur Beurteilung der elterlichen Kapazitäten in Familien mit häuslicher Gewalt: Eine randomisierte kontrollierte Studie

Eine Beurteilung der elterlichen Kapazitäten (Parenting Capacity Assessments; PCAs) ist für die Kinder‐ und Jugendhilfe unerlässlich, um Entscheidungen zur Unterbringung von Kindern zu treffen, die von häuslicher Gewalt betroffen sind. Trotzdem sind derzeit keine evidenzbasierten PCA‐Protokolle verfügbar. In dieser randomisierten kontrollierten Studie haben wir die Qualität eines bindungsbasierten PCA‐Protokolls untersucht, das auf einer Video‐Feedback‐Intervention basiert, um positive Erziehungskompetenz und sensibles Maßregeln (Positive Parenting and Sensitive Discipline; VIPP‐SD) zu fördern. Wir haben 56 Eltern‐Kind‐Dyaden (mittleres Alter der Kinder = 3,48 Jahre) in niederländischen Familienheimkliniken rekrutiert, in denen PCAs durchführt werden, um Entscheidungen zur Unterbringung von Kindern zu treffen. Nach dem Prä‐Test wurden die Familien randomisiert und erhielten entweder das reguläre Beurteilungsverfahren (Regular Assessment Procedure; RAP) (n = 28) oder eine zusätzliche Beurteilung basierend auf VIPP‐SD (n = 28). Nach der Beurteilung folgte ein sofortiger Post‐Test und nach 10 Monaten ein Follow‐up. Multilevel Modelle ergaben, dass sich die Therapeut_innen hinsichtlich ihrer Empfehlungen zur Unterbringung der Kinder in beiden Gruppen gleichermaßen sicher fühlten und dass sie ihre erste Unterbringungsempfehlungen gleichermaßen häufig revidierten. Darüber hinaus zeigten Kinder in der VIPP‐SD‐Gruppe nicht weniger Verhaltensprobleme und erlebten nicht seltener wiederkehrende Misshandlungen als Kinder in der RAP‐Gruppe. Daher fanden wir keine Hinweise darauf, dass PCAs, die das VIPP‐SD‐Protokoll enthalten, gegenüber den üblichen PCAs einen Vorteil haben. Mögliche Erklärungen, warum VIPP‐SD in der vorliegenden Studie die Qualität des RAP nicht zu verbessern schien, werden diskutiert.

## 抄録

虐待する家族の育児能力の評価におけるアタッチメントに基づく介入の効果:ランダム化比較試験

育児能力評価(PCAs) は、児童保護サービスにとって、虐待する家族に対しての 配置決定を裏付けるために必要不可欠であるが、現在エビデンスに基づくPCAプ ロトコルはない。本研究のランダム化比較試験において、我々は、“積極的な子 育てと繊細なしつけを促進するためのビデオ・フィードバック介入(VIPP‐SD)”を 基にしたアタッチメントに基づくPCAプロトコルの質をテストした。我々は、配 置決定を裏付けるPCAsを実施しているオランダの家族滞在クリニックで、56組の 親子 (子どもの平均年齢:3.48歳) を採用した。事前テスト後、家族は通常の評 価手順(RAP) (n = 28)を受けるか、VIPP‐SDを基にした追加の評価(n = 28)を受 けるかを、無作為に選ばれた。直後のテストと10ヶ月のフォローアップが実施さ れた。マルチレベルモデルは、セラピストが両グループとも子どもの配置に関す る自分たちの提案に等しく自信を感じていること、また自分達の最初の配置提案 を等しい頻度で変更していることを示していた。さらに、VIPP‐SD グループの子 ども達は、RAPグループの子ども達より、より少ない行動問題を示すということ はなく、またより少ない児童虐待の再発を体験しないということもなかった。以 上のように、VIPP‐SD プロトコルを取り入れたPCAs が、通常のPCAs より優れて いるという証拠は見られなかった。本研究においてVIPP‐SDがRAPの 質を向上さ せるように見られなかった理由について考えられる解釈を議論する。

## 摘要

尽管育儿能力评估 (PCAs) 对儿童保护服务至关重要, 以支持虐待型家庭的安置决策, 但目前还没有循证PCA方案。在这项随机对照试验中, 我们测试了基于“积极养育和敏感管教的视频反馈干预 (VIPP‐SD)”的PCA干预方案的质量。我们在荷兰家庭寄宿诊所招募了56对母婴 (儿童平均年龄 = 3.48) 进行PCAs以支持安置决策。经过预测试后, 家庭被随机分配以接受常规评估程序 (RAP) (n = 28), 或根据VIPP‐SD (n = 28) 进行额外评估。开展了一次即时后测和10个月随访。多层次模型显示, 治疗师对两组儿童安置的建议同样有信心, 而且他们同样经常修改最初的安置建议。此外, 与RAP组相比, VIPP‐SD组的儿童没有表现出更少的行为问题, 也没有经历反复的儿童虐待。因此, 我们没有发现任何证据表明, 采用VIPP‐SD方案的PCAs的质量优于常规PCAs。我们讨论了可能的解释, 为什么在目前的研究中VIPP‐SD似乎没有增加RAP的质量。

## ملخص

التعلق وتنظيم الإجهاد عند الأطفال المحرومين اجتماعيا ً واقتصادياً: هل تقدم رعاية الأطفال العامة التعويض المطلوب؟

الأطفال الذين ينشأون في أسر تعاني من الحرمان الاجتماعي والاقتصادي (SED) هم أكثر عرضة للعجز في التعلق وتنظيم الإجهاد مقارنة بأقرانهم في الأسر ذات الموارد الاجتماعية والاقتصادية الأكثر. افترضت هذه الدراسة أن مقدمي الرعاية في الرعاية العامة للأطفال قد يساعدون هؤلاء الأطفال على التعويض. ولذلك قمنا بالتحقيق مع 60 طفلاً (30 طفل من الفئات المحرومة اجتماعيا واقتصاديا و30 طفل من الطبقة المتوسطة) وقيمنا تعلق الأطفال(AQS) بالأم ومقدم الرعاية الأولية في مراكز رعاية الطفل العامة. وعلاوة على ذلك، تم قياس إيقاع الكورتيزول اليومي للأطفال على أساس 12 عينة من اللعاب تؤخذ عبر ثلاثة أيام في الأسبوع. وأظهر الأطفال المحرومون درجات أقل على مقياسAQS مع أمهاتهم مقارنة بمقدمي الرعاية. بالمقارنة مع نظرائهم، أظهر الأطفال المحرومون أيضًا إفراز كورتيزول متزايد وملامح الكورتيزول التملقية التي تعكس أنشطةHPA العالية بشكل عام وقدرات أقل لتنظيم الإجهاد. وأبرزها ومع ذلك، ربط نمذجة المسار متعدد المستويات أعلى مستوى الرعايةAQS عشرات لخفض الإفراج الكورتيزول على مدار الأسبوع.

## 1 INTRODUCTION

Child maltreatment constitutes a major public health concern; it affects millions of children worldwide and is associated with a broad spectrum of negative and long‐lasting developmental outcomes (Gilbert et al., 2009). When child maltreatment is suspected or substantiated in a family, child protection services may consider out‐of‐home placement. Essential for deciding whether or not a child should be placed out of home are assessments of parenting capacities (PCAs). Unfortunately, currently no evidence‐based methods for PCAs are available. Considering the complexity of placement decisions and their impact on the lives of children and their parents, valid PCA protocols are needed to effectively support placement decisions. One proposal for improved PCA protocols is that parenting capacities should be evaluated based on parents’ response to an evidence‐based intervention (Harnett, 2007). Building on this proposal and existing theories regarding child maltreatment and its etiology, several researchers have suggested using an attachment‐based intervention for this purpose (Cyr & Alink, 2017; Cyr et al., 2012; Lindauer, Bakermans‐Kranenburg, Van IJzendoorn, & Schuengel, 2010). Parallel to a recent Canadian study (Cyr, Paquette, Dubois‐Comtois, & Lopez, 2015), the current randomized controlled trial (RCT) is among the first to empirically evaluate whether the quality of placement decisions can be improved by structurally evaluating parents’ response to an attachment‐based intervention.

### 1.1 Parenting capacity assessments

Although a number of guidelines have been developed for PCAs (e.g., American Psychological Association, 1998; Budd, 2001), empirical evidence on the effectiveness of these assessments is scarce (Vischer, Grietens, Knorth, & Mulder, 2017). In addition, several limitations of PCAs in practice have been reported: these assessments often concern only one time point, do not include observations of parent‐child interactions in the home environment, and emphasize parents’ weaknesses more than their strengths (Budd, 2001, 2005). In order to improve the quality of these assessments, several researchers have suggested using a more structured and dynamic approach (Cyr et al., 2012; Harnett, 2007; Lindauer et al., 2010). The approach they propose consists of structurally assessing parents’ capacity to change relevant parenting behavior by evaluating parents’ response to an evidence‐based intervention. The intervention should be conducted in a short time period, include systematic observations of the parent‐child relationship in the home setting, and focus on the strengths of parents.

Two things should be noted with respect to the implementation of this assessment approach. First, such an approach would be particularly valuable for cases that are equivocal and where an initial (risk) assessment does not demonstrate a clear picture of the child's well‐being (Harnett, 2007). Second, it should be emphasized that information about parents’ response to a parenting intervention should always be integrated with other relevant risk and protective factors in the family and be interpreted within this context. For instance, if improved quality of parent‐child interactions is observed, but the mother continues to have a violent relationship with her partner, this should also be taken into account in the PCA.

### 1.2 Focus on attachment‐based interventions

Based on the existing knowledge on maltreatment, parents’ response to an attachment‐based intervention aimed at improving parental sensitivity would provide highly relevant information for a PCA (Cyr & Alink, 2017; Cyr et al., 2012; Lindauer et al., 2010). Parental sensitivity, which refers to parents’ ability to notice, interpret, and respond to child signals in an appropriate and prompt manner while adapting to the child's changing developmental needs (Ainsworth, Bell, & Stayton, 1974), is universally considered as an important indicator of positive child development (Ainsworth et al., 1974; Mesman, Van IJzendoorn, & Bakermans‐Kranenburg, 2012) and has often been identified as relevant for PCAs (Cyr & Alink, 2017; Cyr et al., 2012; Lindauer et al., 2010; Teti & Candelaria, 2002; Ward, Brown, & Hyde‐Dryden, 2014; White, 2005).

Several studies have shown that attachment‐based interventions aimed at improving parental sensitivity have positive effects for maltreating parents, or parents at risk for maltreatment, and their children (Bernard et al., 2012; Moss et al., 2011; Negrao, Pereira, Soares, & Mesman, 2014; Steele, Murphy, Bonuck, Meissner, & Steele, 2019). These studies found positive outcomes both at the level of the parent‐child relationship (i.e., increased quality of parental sensitivity and the attachment‐relationship and less harsh discipline) and at the level of child development (i.e., improved self‐regulation skills and fewer behavioral and emotional problems). Besides their focus on improving parental sensitivity, these interventions have in common that they are short‐term, include videofeedback, and focus on parents’ strengths. The effectiveness of these interventions has been strongly supported by empirical evidence, which increases the informational value of response to intervention or lack thereof (Cyr et al., 2012; Harnett, 2007; Lindauer et al., 2010). A recent Canadian study found that implementing a PCA protocol based on an evidence‐ and attachment‐based video‐feedback intervention enabled clinicians to better predict reoccurrences of child maltreatment (Cyr et al., 2015). Although these results are promising, more studies are necessary, (1) to establish these effects more firmly, and (2) to evaluate whether such a protocol could also be effective in other countries with different child protection systems.

**Key findings**

1. An attachment‐based parenting intervention, such as VIPP‐SD, can be used to conduct a dynamic assessment of parenting capacities in child protection cases involving young children. This could contribute to improved decision‐making.
2. In this study, we found no credible evidence to suggest that parenting capacity assessments incorporating VIPP‐SD outperformed parenting capacity assessments as usual.
3. These findings do not prove that using VIPP‐SD for parenting capacity assessments does not affect the quality of decision‐making, but do show that if VIPP‐SD has any effect, it is likely to be smaller than we had expected a priori and could therefore not be detected with the current, relatively small, sample. Which effect size would be of clinical interest relative to the costs involved for this procedure awaits further discussion, which should inform the design and the size of future trials.

**Relevance to the field of Infant and Early Childhood Mental Health**

This study evaluated the effectiveness of an attachment‐based parenting capacity assessment protocol for maltreating families for whom a placement decision was being considered. More knowledge on this topic can contribute to improved decision‐making in child protection cases involving young children.

### 1.3 Evaluating the quality of placement decisions

The quality of a procedure for PCAs depends on the reliability and validity of subsequent placement decisions. Relating this to the current study, the reliability of the proposed assessment approach has been recently investigated in a vignette study where we demonstrated that providing decision‐makers with information about parents’ response to an attachment‐based intervention can lead to increased agreement on placement decisions (Van der Asdonk et al., 2019). This is an important foundation for the current study, because sufficient reliability is a prerequisite for strong validity. Although validity might be a difficult construct to appropriately evaluate in this context, improved validity of placement decisions should at least be reflected in (a) professionals’ confidence that their recommendation regarding the child's placement is accurate (face validity) and (b), because the main goal of child protection services is to act in the best interest of children's well‐being, an improved quality of life for children (predictive validity).

Importantly, several longitudinal studies have shown that reunifications of maltreated children with their parents are often not stable over time and that some parents will abuse or neglect their children again in the future (Biehal, Sinclair, & Wade, 2015; Lutman & Farmer, 2013). This indicates that severe parenting problems may still exist and children's quality of life does not always improve following placement decisions. Moreover, mixed results have been reported regarding children's mental well‐being, with some studies showing worse outcomes for children who were reunified with their parents than for children who remained in out‐of‐home care (Biehal et al., 2015), and other studies finding opposite results (Lloyd & Barth, 2011). These findings do not only emphasize the complexity of placement decisions, but also stress the need for studies that take children's well‐being into account when evaluating methods to improve the quality of decisions. Therefore, in the current study we looked at reoccurrences of child maltreatment and children's emotional and behavioral problems as indicators of their quality of life following placement decisions. In addition, we looked at the severity of parenting problems for birth parents following placement decisions as a proxy of children's well‐being.

### 1.4 Reasoning biases in decision‐making

One aspect that has been found to compromise the quality of decision‐making is related to common reasoning biases in decision‐making (Kahneman, Slovic, Slovic, & Tversky, 1982). In a study that investigated professional reasoning in child protection reports, it was shown that professionals can be prone to hold on to their initial judgments about a family, even when they are faced with new and contradictory evidence (Munro, 1999). One way to prevent such intuitive reasoning mistakes might be to provide more concrete, relevant, and objective information for professionals to guide their decision‐making. Such concrete information may be produced by a structured, attachment‐based assessment protocol (Cyr et al., 2012; Lindauer et al., 2010), because it informs professionals about parents’ ability to benefit from an intervention to improve important parenting skills. If this information can indeed reduce reasoning biases in child protection cases, this should be reflected by a higher tendency of professionals to change their initial judgments after receiving additional information provided by the assessment protocol.

### 1.5 Current study

The current RCT tested the effect of evaluating parents’ response to an attachment‐based intervention on the quality of placement decisions in the Netherlands. For this purpose, we developed a procedure for PCAs based on the Video‐feedback Intervention to promote Positive Parenting and Sensitive Discipline (VIPP‐SD; Juffer, Bakermans‐Kranenburg, & Van IJzendoorn, 2017), an assessment procedure that is similar to the protocol developed by Cyr et al. (2012). We hypothesized (1) that recommendations about the necessity of out‐of‐home placement at the start of families’ assessment period were more often modified by therapists after VIPP‐SD than after the regular assessment procedure (RAP), (2) that therapists felt more confident on their recommendations based on VIPP‐SD than on their recommendations based on the RAP, (3) that children for whom a recommendation was based on VIPP‐SD showed fewer emotional and behavioral problems than children for whom a recommendation was based on the RAP, and (4), for the group of children who returned to their parents after the assessment, that there were fewer reoccurrences of child maltreatment in families for whom a recommendation was based on VIPP‐SD than in families for whom a recommendation was based on the RAP. In addition to these primary research questions, we explored whether the evaluation of parenting capacities differed between families who received VIPP‐SD and families in the RAP. Finally, for the group of children who returned to their parents after the assessment, we explored whether families for whom a recommendation was made based on VIPP‐SD received less intensive parenting support, indicating less severe parenting problems, after leaving the clinic than families for whom a recommendation was made based on the RAP.

## 2 METHODS

### 2.1 Sampling procedure

Recruitment took place from May 2015 until December 2017 in four family residential clinics that are located in different regions of the Netherlands. These clinics constitute a unique setting in the Dutch child protection system which enables highly intensive observation and treatment of families for whom a placement decision is being considered (either in the context of an out‐of‐home placement or a reunification). Families usually reside in these clinics for 24 h a day on weekdays (and, if necessary, during weekends) for a period of 2 to 3 months, during which they are regularly observed by family workers and receive highly intensive support at all levels of the family system. The evaluation of families’ trajectory at the clinics is used as a recommendation for the children's court judge or involved family guardian, depending on who referred the family to the clinic.

**FIGURE 1:**
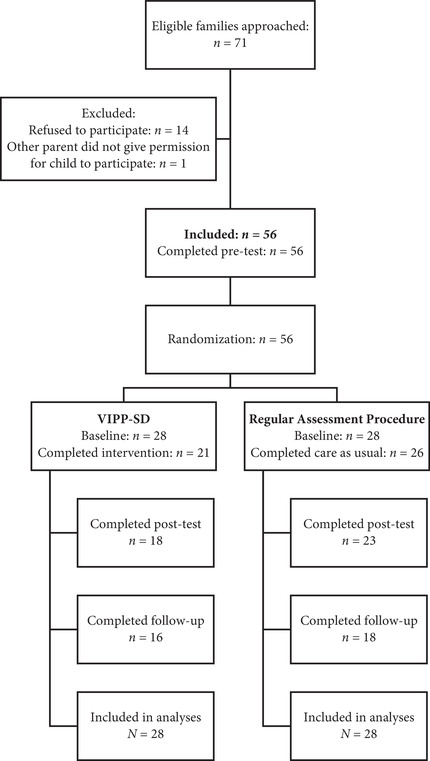
Flow chart of study sample throughout the RCT

For the current study, families were selected based on the following inclusion criteria: (1) the family was referred to the clinic for an evaluation of their parenting capacities in the context of a decision regarding out‐of‐home placement or reunification with their child(ren), (2) the child's age was between 6 months and 7 years, (3) the primary caregiver spoke a basic level of Dutch, (4) the primary caregiver did not have a (severe) intellectual disability that affected his or her ability to understand the instructions of the intervention, and (5) the primary caregiver did not have severe mental health problems which required acute intervention. If a family that met our inclusion criteria started their assessment in the clinic, one of the staff members informed the researchers so that they could explain the study to the families. The recruitment goal was set on 71 families. A power analysis in G*Power 3.0 (Faul, Erdfelder, Lang, & Buchner, 2007) conducted prior to this study indicated that for 60 randomized participants and two‐tailed significance tests at α = .05, power to detect medium effects of *f* = 0.30 on primary study outcomes would be .80. The majority of approached families (79%) agreed to participate. We asked the primary caregiver to participate. If there was more than one child in the family, the youngest child between 1 and 7 years was invited to participate. Overall, 41 families (73% of enrolled families) completed the post‐test. All families, except for those who indicated they did not want to participate anymore (*n* = 6), were approached again for follow‐up. The final follow‐up sample consisted of 34 dyads (61% of the original sample). See Figure 1 and Appendix A for a more detailed description of the sample flow.

### 2.2 Sample

Fifty‐six families participated in this study. About half of the children (55%) were boys, and the children were on average 3.48 years old (*SD* = 1.74). Primary caregivers had an average age of 32.32 years (*SD* = 6.43) and were primarily mothers (93%). Most parents were single parents (64%). For 55% of the families, referral to the clinic concerned assessment regarding a possible reunification with the participating child. The families stayed in the clinic for on average 12.09 weeks (*SD* = 5.49; range: 2‐28 weeks). Families who received VIPP had completed on average 4.36 sessions (*SD* = 2.00; range: 0‐6 sessions). Families’ case records at the clinic were coded for type of maltreatment that had occurred within the participating parent‐child dyad prior to their admission to the clinic based on the Modified Maltreatment Classification System (MMCS; English, Bangdiwalab, & Runyan, 2005). Two trained researchers coded all available files (*κ* = .86 for physical neglect, *κ* = .84 for emotional neglect, *κ* = .68 for physical abuse, *κ* = .84 for emotional abuse; *n* = 15). For six families, no recordings were available at the time of coding. Physical abuse had occurred in 24% of the dyads (*n* = 12), emotional abuse in 20% (*n* = 10), physical neglect in 28% (*n* = 13), and emotional neglect in 73% (*n* = 33). Sexual abuse had occurred in one dyad. Comorbidity was found for 26% of the dyads (*n* = 11). For three dyads, no maltreatment could be substantiated based on the file recordings. These numbers likely underrepresent the actual presence of maltreatment, because available information was often vague or incomplete.

### 2.3 Procedure

This research was approved by the Dutch Central Committee on Research Involving Human Subjects, the ethical review board of the Institute of Education and Child Studies at Leiden University, and the Ethics Committee for Legal and Criminological Research at Vrije Universiteit Amsterdam. The study is registered in the Netherlands Trial Register (Trial NL7632). The pretest was conducted as soon as was possible after the parent(s) signed informed consent for the study and consisted of a 2‐h appointment in a lab setting at the clinic. In addition, the therapist responsible for the family's recommendation on future placement filled out a short questionnaire about the family. After pretest, families were randomly assigned to either VIPP‐SD (*n* = 28) or the RAP (*n* = 28). Randomization was done by one of the researchers with a computer‐generated blocked randomization sequence that was stratified for the four clinics, so that for each clinic the families were equally divided over the two conditions. The post‐test was similar to the pretest and took place on average 9.5 weeks after pretest. Initially, we aimed to conduct two follow‐up assessments at 8 and 12 months. However, due to practical issues making it complicated to complete two follow‐up assessments with the families (i.e., phone numbers changed, multiple efforts required to reach parents at their homes for one appointment) it was decided to conduct only one follow‐up assessment for each family. This assessment took place approximately 10 months after post‐test (range: 8‐16 months) and consisted of a 1‐h home visit during which the primary caregiver filled out questionnaires and a semi‐structured interview was conducted by a trained researcher. Four participating children lived in a foster family at the time of the follow‐up assessment. To contact the foster family, parental permission was required, which was granted for two of these children. Foster parents were asked to fill out questionnaires through email–preceded by a telephone appointment to provide information on the study and to request informed consent. After pre‐ and post‐test, families received a gift card of 20 euros, and after follow‐up they received a small gift.

### 2.4 Assessment of parenting capacities

#### 2.4.1 Regular Assessment Procedure

The RAP consisted of care as usual at one of the clinics. Although the four clinics were not completely uniform in their treatment programs, the general structure was similar: all parents received various forms of treatment aimed at improving family dynamics, including observations of parent‐child interactions, group sessions with other parents, and individual sessions for the parent(s). Some parents and children additionally received specialized therapy based on their individual needs (e.g., trauma therapy or emotion‐regulation training). To limit similarities with VIPP‐SD, none of the families in the RAP condition received video feedback. The standard period for family treatments differed between the clinics (range: 8‐12 weeks). In all clinics, an evaluation was conducted at the end of the assessment period in which the therapist and involved family workers evaluated parents’ progress during their treatment in the clinic. This evaluation resulted in a recommendation that was provided to either the involved family guardian, social worker, or children's court judge, depending on who had referred the family to the clinic.

To be able to use the content of this evaluation for research purposes, we composed a structured parenting capacity evaluation form and asked the therapists to fill out this form for each family at the end of the family's assessment period. This form consists of 15 items on a six‐point Likert‐scale, of which five items concern general aspects of the therapeutic relationship and parents’ attitude during the intervention (e.g., *Was the parent open to change his/her behavior?*), and ten items concern changes in parents’ behavior following the intervention (e.g., *The parent shows progress in adequately responding to negative child signals, such as crying and resistant or naughty behavior*). The internal consistency of the assessment form was high (α for all 15 items = .93). In each clinic, there was one therapist (with a Master degree) who was responsible for families’ recommendations. The family workers generally had a Bachelor degree and worked directly with the families.

#### 2.4.2 VIPP‐SD

We slightly adapted VIPP‐SD by adding an explicit evaluation of parenting capacities at the end of the intervention. Thus, VIPP‐SD in this study consisted of (1) an intervention and (2) an assessment form. For the intervention part, either VIPP or VIPP‐SD (Juffer, Bakermans‐Kranenburg, & Van IJzendoorn, 2008) was delivered to the family, depending on the child's age: parents of a 6‐ to 12‐months‐old received VIPP (*n* = 3) and parents of a child older than 12 months received VIPP‐SD. VIPP focuses on improving parental sensitivity through video feedback and consists of six sessions in which the parent‐child dyad is videotaped during common, daily interactions such as playing together or a meal. VIPP‐SD additionally focuses on improving sensitive discipline strategies of the parent. For a detailed overview of VIPP(‐SD), see Juffer et al. (2008). VIPP‐SD was delivered by family workers at the clinics who were trained to be VIPP‐interveners for this study. For six parent‐child dyads, a trained (assistant) researcher provided VIPP‐SD because no trained family worker was available at that time. Each VIPP‐SD trajectory was monitored during supervision meetings with one of the trained researchers. After the final session, we asked the intervener to fill out the parenting capacity assessment (PCA) form to evaluate parents’ response to VIPP‐SD and to integrate the assessment form in the evaluation of the family at the end of their treatment period (as described above in the RAP section). Finally, similar to the RAP group, we asked the therapists to fill out an evaluation form for their recommendation regarding the child's placement.

### 2.5 Instruments

#### 2.5.1 Recommendation regarding child placement

At pre‐ and post‐test, we asked the therapist to indicate the current recommendation for this family: (a) a supervision order, but the child can stay or be reunified with its parent(s), (b) (extended) supervision order and out‐of‐home placement of the child–in own network, (c)–in foster care, (d)–in residential care, or (e) other. We dichotomized these items into (0) no out‐of‐home placement versus (1) out‐of‐home placement.

#### 2.5.2 Therapists’ confidence in their recommendation

After the therapists gave their recommendation about the child's placement at pre‐ and at post‐test, we asked them to indicate on a ten‐point scale how confident they felt about their recommendation. A higher score indicated more confidence.

#### 2.5.3 Children's emotional and behavioral problems

The preschool version of the Child Behavior Checklist (CBCL) was used to assess children's emotional and behavioral problems (Achenbach & Rescorla, 2000). The CBCL consisted of 100 items regarding the child's behavior in the past 2 months which are rated on a three‐point scale (0 = not true, 1 = somewhat or sometimes true, and 2 = very or often true). We asked the primary caregiver to fill out the CBCL at pre‐ and post‐test and at the follow‐up assessment. For two children who lived in foster care at follow‐up, the involved foster parent filled out the CBCL. The CBCL has been proven valid and reliable (Achenbach & Rescorla, 2000). Sum scores for total problems (α in current sample = .98) were used. Because of an extremely high number of missing post‐test scores for the CBCL (71% of the forms were missing, compared to 46% for both pretest and follow‐up), we decided not to use the post‐test data, so that only CBCL scores at pretest and follow‐up were compared.

#### 2.5.4 Recurring child maltreatment

For those children who were living with their parents at follow‐up (*n* = 32; 94%), we assessed whether there had been reoccurrences of child maltreatment in the 10 months that followed leaving the clinic. For this purpose, a trained (assistant) researcher conducted the Maternal Maltreatment Classification Interview (MMCI; Cicchetti, Toth, & Manly, 2003) with the primary caregiver. The MMCI is a semistructured interview during which the primary caregiver is asked about events of child abuse and the family's contact with child protection services. We used the version that was translated into Dutch by Reijman et al. (2014). We asked the primary caregiver to answer the questions about the 10 months after they had left the clinic. The MMCI was coded using the MMCS (English, Bangdiwalab, & Runyan, 2005). After coding, each family received a score reflecting whether child maltreatment had reoccurred (1) or not (0). Two trained (assistant) researchers double‐coded all interviews, reliability was excellent (κ = 1.00, *n* = 28).

#### 2.5.5 Intensity of parenting support at follow‐up

During the MMCI with the biological primary caregiver at follow‐up, we additionally asked about the involvement of professional care specifically aimed at parenting since they left the clinic. We coded their answers on a seven‐point scale, ranging from (0) no extra care (other than standard post‐treatment care), to (6) parenting support is currently present more than once per week. All interviews were independently coded by two trained coders, reliability was high (Intraclass Correlation Coefficient [ICC], single measures = .98, *n* = 26).

#### 2.5.6 Evaluation of parenting capacities

We used the PCA form that was developed for this study to get an indication of parents’ capacities following VIPP‐SD or RAP as evaluated by the involved therapist or VIPP‐intervener (for a more detailed description of this form, see Procedure section of this article). Higher average scores across the 15 items in the analyses indicated that the involved therapist or intervener evaluated the parent as more capable.

### 2.6 Statistical analyses

#### 2.6.1 Missingness

Data inspection revealed that the numerical variables approached a normal distribution after winsorizing outliers more than ±3.29 standard deviations from the mean. One family could only be reached for follow‐up after 23 months. For this family, we decided to still use the data retrieved from the MMCI (recurring maltreatment and intensity of parenting support), as the interview specifically aimed at the first 10 months after leaving the clinic. CBCL scores for this family were not used, because this construct is likely more difficult to rate objectively in retrospect. Little's MCAR test (Little, 1988) showed that values were missing completely at random (*χ* ^2^ (138) = 139.97, *p* = .44). To follow an intent‐to‐treat approach and maximize power, multilevel multiple imputation (Rubin, 1987; White, Carpenter, & Horton, 2012) was performed on the data (*N* = 56) in RStudio (version 1.1.463) (RStudioTeam, 2016). See Supporting information Appendix B for a detailed overview of imputation procedures.

#### 2.6.2 Main analyses

For therapists’ confidence in their recommendation and children's behavioral and emotional problems, three‐level linear mixed effect models accounting for repeated measures over time (level 1) and nesting of families (level 2) within clinics (level 3) were incrementally compared using likelihood ratio test for imputed datasets in the *mitml* package. The final model included the fixed effects of time (coded as 0 = pretest, 1 = post‐test/follow‐up), the main effect for condition (coded as 0 = RAP, 1 = VIPP‐SD), and the interaction between time and condition. For modifications in therapists’ recommendation regarding child placement, a similar model was fitted with a binomial family structure. For recurring maltreatment, generalized linear mixed effect models accounting for nesting of families (level 1) in clinics (level 2) were performed with the *lme4* package with a binomial family structure. We compared models incrementally with likelihood ratio tests. We explored the influence of two potential covariates: (1) time between post‐test and follow‐up (because of the large range in time) and (2) children's age (because of the relatively broad age range in our study). However, because neither of these covariates affected any of the results, we reported only the most parsimonious models without covariates. After testing our main hypotheses, we explored potential differences between VIPP‐SD and RAP families in the evaluations of their parenting capacities at post‐test and in the intensity of parenting support at follow‐up. For this purpose, we compared two linear mixed effect models accounting for the nesting of families within clinics. Significance of model and parameter estimates was determined at α = .05. Complete case analyses yielded similar outcomes (available upon request). Odds ratios were computed as estimates of effect sizes for dichotomous outcome variables (i.e., modifications in therapists’ recommendation regarding child placement and recurring child maltreatment), and beta's were used as estimates of effect sizes for continuous outcome variables (see e.g., Lorah, 2018).

#### 2.6.3 Equivalence tests

We performed equivalence tests to evaluate whether effects that failed to reach statistical significance were caused by insufficient statistical power to test for the a priori expected effect size (Lakens, 2017). For this purpose, we followed a multilevel procedure for two one‐sided tests described by Isager (2019) for the linear mixed effect models (on complete case analyses, because this procedure is not applicable for multilevel imputation analyses). Lower and upper bounds were set to the a priori established effect size for this study (*d* = 0.60), which was converted to a raw effect size for each of the analyses. Because to our knowledge, there is presently no such procedure that is applicable to generalized linear mixed models, we did not perform equivalence tests for these models.

## 3 RESULTS

### 3.1 Preliminary analyses

For an overview of demographic and outcome variables of the total sample, see Table 1. The majority of children (88%) were living with their parent(s) at the follow‐up assessment. For 94% of the children, their living situation at follow‐up was consistent with the final recommendation the family received in the clinic. There were no differences between the VIPP‐SD and RAP groups at pretest variables (see Table 1). Moreover, comparisons on demographic and target variables between families who dropped out during the research project and families who completed the project showed that there were no significant differences (*p's* >.10). Pooled correlations between all variables of interest are displayed in Table 2.

**TABLE 1:** Descriptive statistics for demographic and target variables

	All families		VIPP‐SD		Regular Assessment		
	*M* a	*SD* a	*M* a	*SD* a	*M* a	*SD* a	*F/*χ^2^ b
Demographic	*N = 56*		*n =* 28		*n =* 28		
Age child	3.48	1.74	3.78	1.88	3.19	1.57	1.04
Gender child (% boys)	55%		54%		57%		0.07
Age parent	32.32	6.43	33.69	7.10	31.50	7.35	0.20
Gender parent (% female)	93%		93%		93%		<0.00
Number of siblings	2.52	1.39	2.44	1.67	2.61	1.09	0.26
Outcome variables at Pretest							
Therapists’ recommendation (% out‐of‐home placement)	50%		46%		54%		0.35
Therapists’ confidence	6.07	1.57	5.91	1.51	6.08	1.69	0.66
CBCL total	36.56	28.87	36.66	29.45	36.47	29.27	0.16
Outcome variables at Post‐test	*n* = 41		*n* = 18		*n* = 23		
Therapists’ recommendation (% out‐of‐home placement)	29%		36%		22%		
Therapists’ confidence	7.74	1.04	7.92	0.97	7.59	1.10	
Evaluation of parenting capacities	4.04	0.75	3.80	0.83	4.30	0.57	
Outcome variables at Follow‐up	*n* = 34		*n* = 16		*n* = 18		
CBCL total	36.92	22.43	44.23	25.43	31.32	18.71	
Recurring maltreatment (*N*)	*n =* 9		*n =* 5		*n =* 4		
Intensity of parenting support	4.02	2.22	3.15	2.26	4.56	2.07	

a Unless indicated otherwise.

b Chi‐square tests and one‐way ANOVA's were performed to test whether there were pre‐test group differences between families in the VIPP‐SD and RAP conditions.

**TABLE 2:** Pooled Pearson Correlations between study variables of interest (*N* = 56)

	2	3	4	5	6	7	8	9	10	11	12
Pretest variables											
1. Age child	.12	.20	−.14	−.12	.04	−.02	−.06	−.09	.14	.19	−.04
2. Gender child^1^		.03	−.06	.07	−.04	.21	.01	.02	.00	−.09	−.02
3. Age parent			.14	−.12	.10	.16	−.04	−.18	.27	.20	.18
4. Therapists’ recommendation^2^				.16	.09	.33*	−.06	−.26	−.01	.11	.27
5. Therapists’ confidence					.03	.32*	.18	.01	−.07	−.09	.11
6. CBCL total						.06	−.07	.06	.28	.05	.15
Post‐test variables											
7. Therapists’ recommendation^2^							.03	−.40*	.03	−.06	.08
8. Therapists’ confidence								.03	−.02	−.04	−.20
9. Evaluation of parenting capacities									.01	−.05	−.04
Follow‐up variables											
10. CBCL total										.13	.03
11. Recurring child maltreatment											.06
12. Intensity of parenting support											

* *p *< .05.

^1^ Coded as girl = 1.

^2^ Coded as out‐of‐home placement = 1.

### 3.2 Modifications in therapists’ recommendations regarding child placement

For modifications in therapists’ recommendations regarding child placement, the unconditional growth model showed the best fit (see Table 3). Only the fixed effect of time was significant and indicated that compared to pre‐test, therapists’ recommendations at post‐test more often favored that the child could stay with its parents, see Table 1. Recommendations for VIPP‐SD families were not more often modified than recommendations for RAP families.

**TABLE 3:** Fixed effects of linear mixed models, dependent (binomial) variable is therapists’ recommendation regarding child placement (*N* = 56)

	Model 1	Model 2		Model 3		Model 4	
	*B* (SE)	*B* (SE)	*OR*	*B* (SE)	OR	*B* (SE)	OR
Fixed effects							
(Intercept)	0.41 (0.06)*	0.69 (0.14)*		0.67 (0.15)*		0.84 (0.19)*	
Time		−0.19 (0.08)*	0.83	−0.19 (0.08)*	0.83	−0.30 (0.11)*	0.74
Condition				0.04 (0.12)	1.04	−0.30 (0.28)	0.74
Time*Condition						0.23 (0.16)	1.26
Variance components							
Clinic level^1^							
Family level	1.09 (0.31)	1.37 (0.36)		1.36 (0.36)		1.50 (0.44)	
Change in model fit (*F*)		4.52*		0.14		1.76	

Time: 0 = pretest, 1 = post‐test; Condition: 0 = Regular Assessment Procedure, 1 = VIPP‐SD.

* *p* < .05.

^1^ Nesting of families within clinics could not be fitted with this model.

### 3.3 Therapists’ confidence in their recommendation

Therapists’ confidence in their recommendation varied more over time (ICC = .81) and between therapists (ICC clinic level = .14) than between families (ICC = .01). The unconditional growth model including the fixed effect of time showed the best fit and indicated that for both conditions, therapists felt more confident on their recommendation at post‐test than at pretest (see Tables 1 and 4). The fixed effect of the interaction between time and condition was not significant, which indicates that therapists did not feel more confident over time about their recommendations for VIPP‐SD families than about their recommendations for RAP families. The equivalence test was significant (*t* (59.90 = 3.15, *p* <.01), indicating that the size of the effect was statistically equivalent between the two conditions (i.e., the observed effect size was significantly lower than the a priori expected effect size).

**TABLE 4:** Fixed effects of linear mixed models for therapists’ confidence in their recommendation and total CBCL scores (*N* = 56)

	Model 1	Model 2		Model 3		Model 4	
	*B* (SE)	*B* (SE)	β	*B* (SE)	β	*B* (SE)	β
DV: Therapists’ confidence							
Fixed effects							
(Intercept)	6.94 (0.21)*	4.43 (0.48)*		4.33 (0.51)*		4.58 (0.66)*	
Time		1.67 (0.29)*	.73	1.67 (0.29)*	.73	1.50 (0.40)*	.66
Condition				0.19 (0.33)	.06	−0.31 (0.98)	−.16
Time*Condition						0.33 (0.59)	.15
Variance components							
Clinic level	0.27 (0.16)	0.30 (0.16)		0.30 (0.16)		0.29 (0.16)	
Family level	0.01 (0.05)	0.51 (0.22)		0.51 (0.22)		0.51 (0.21)	
Residual	1.62 (0.07)	1.27 (0.09)		1.27 (0.09)		1.28 (0.09)	
Change in model fit (*F*)		29.43*		0.47		0.46	
DV: CBCL total							
Fixed effects							
(Intercept)	38.17 (3.43)*	38.13 (10.00)*		35.24 (10.47)*		44.34 (13.75)*	
Time		0.03 (6.06)	.00	0.03 (6.06)	.00	−6.04 (8.13)	−.17
Condition				5.78 (6.79)	.16	−12.42 (20.41)	−.35
Time*Condition						12.14 (12.15)	.34
Variance components							
Clinic level	0.72 (1.44)	0.72 (1.44)		0.85 (1.64)		0.86 (1.64)	
Family level	12.99 (3.73)	12.97 (3.79)		12.71 (4.00)		13.20 (3.58)	
Residual	22.62 (2.17)	22.61 (2.26)		22.61 (2.26)		22.20 (2.07)	
Change in model fit (*F*)		0.40		0.82		1.35	

Time: 0 = pretest, 1 = post‐test (for therapists’ confidence) or follow‐up (for CBCL) Condition: 0 = Regular Assessment Procedure, 1 = VIPP‐SD.

* *p* < .05.

### 3.4 Behavioral and emotional problems

Children's behavioral and emotional problems varied more over time (ICC = .61) and between families (ICC = .36) than between clinics (ICC = .03). Adding fixed effects to the unconditional means model did not improve model fit (see Table 4). This indicates that generally, children did not change over time in their level of behavioral and emotional problems. Moreover, even though children who received a placement decision after participating in VIPP‐SD showed an increase in behavioral and emotional problems over time whereas children who received RAP showed a decrease over time (see Table 1), this difference in change was not statistically significant from zero. The equivalence test was not significant (*t*(51.94) *=* 1.08, *p* = .28), which indicates that the size of the effect was not statistically equivalent (i.e., the observed effect size was not significantly different from the a priori expected effect size). Thus, the effect can be considered as undetermined because there is not enough data to draw conclusions (Lakens, 2017).

### 3.5 Recurring child maltreatment

For recurring child maltreatment, the unconditional means model showed the best fit, see Table 5. This indicates that there were no differences in experienced recurring child maltreatment between children in the VIPP‐SD group and children in the RAP group.

**TABLE 5:** Fixed effects of generalized linear mixed models, dependent variable is recurring child maltreatment (*N* = 56)

	Model 1	Model 2	
	*B* (SE)	*B* (SE)	OR
Fixed effects			
(Intercept)	0.35 (0.09)*	0.30 (0.12)*	
Condition		0.10 (0.17)	1.11
Variance			
Clinic level	0.22 (0.24)	0.23 (0.24)	
Change in model fit (*F*)		0.32	

Condition: 0 = Regular Assessment Procedure, 1 = VIPP‐SD.

* *p* < .05.

### 3.6 Exploratory analyses

For the evaluation of parenting capacities at post‐test, the fixed effect of condition improved model fit compared to the empty model (*F*(1, 1012.76) = 5.25, *p* = .02; *B* = −0.51, β = −.48, *SE* = 0.23, *p* = .02). The fixed effect estimate indicates that on average, families in the VIPP‐SD group were evaluated as less capable than families in the RAP group (see Table 1). The size of this effect was not statistically equivalent (*t*(36.09) = −0.16, *p* = .87), indicating that the observed effect size was not significantly different than the a priori expected effect size.

With respect to the intensity of parenting support at follow‐up, VIPP‐SD families received less intensive parenting support at follow‐up than RAP families (see Table 1), although this difference was not statistically different from zero (*F*(1, 255.89) = 1.88, *p* = .17; fixed effect for condition: *B* = −1.05, β = −.33, *SE* = 0.75, *p* = .17). The size of this effect was not statistically equivalent (*t*(22.11) = −0.13, *p* = .90). Thus, the observed effect size was not significantly different from the a priori expected effect size, which implicates that more data would be required to draw a conclusion regarding this effect.

## 4 DISCUSSION

PCAs are an important basis for placement decisions, although thus far no evidence‐based methods for this purpose are available. This study was among the first to investigate through an RCT whether the quality of placement decisions for maltreating families could be improved by implementing a structured, attachment‐based PCA. We investigated this in four Dutch family residential clinics that conducted PCAs in the context of a potential out‐of‐home placement decision–a setting that is unique in the Dutch child protection system. In addition to the RAP, half of the families received an assessment based on VIPP‐SD, an attachment‐based video‐feedback intervention (Juffer et al., 2017). We evaluated the quality of the assessment procedures in terms of face validity (therapists’ confidence that their recommendation regarding the child's placement was accurate) and predictive validity (children's change in well‐being after a placement decision). In addition, we hypothesized that therapists would be more reluctant to change their initial recommendations for families who received a RAP than for families who received VIPP‐SD. None of our hypotheses were confirmed in this study: therapists did not feel more confident about their recommendations for families whose assessment was based on VIPP‐SD, neither did they modify their initial recommendations more often for families who received an assessment based on VIPP‐SD than for families who received the RAP. Moreover, children in families who received an assessment based on VIPP‐SD did not differ from children in families who received the RAP with respect to (a) their level of problem behavior and (b) their chance of experiencing recurring child maltreatment in the 10 months following the placement decision.

To evaluate whether the absence of statistically significant findings in this study could be attributed to power issues, we performed equivalence tests (Lakens, 2017). For therapists’ confidence in their placement recommendations, the equivalence test showed that the observed effect size was smaller than our a priori established effect size of *d* = 0.60. Although equivalence tests could not be performed for all main analyses, the observed effect sizes for both modifications in therapists’ recommendations (OR = 1.26) and recurring child maltreatment (OR = 1.11) were also quite small. Therefore, these findings do not prove that using VIPP‐SD for PCA does not affect the quality of decision‐making, but do suggest that if VIPP‐SD has any effect, it is likely to be smaller than we had expected a priori and could therefore not be detected with the current, relatively small, sample. For instance, our a priori expected effect size of *d* = 0.60 might have been rather optimistic for this study. It could be argued that in the context of out‐of‐home placement decisions, smaller effect sizes can also be considered as clinically relevant (e.g., Lakens, 2013). Which effect size would be of clinical interest relative to the costs involved for this procedure awaits further discussion, which should inform the design and the size of future trials.

In addition to our main hypotheses, we explored whether there were differences between families who received VIPP‐SD and families in the regular assessment group in the evaluation of their parenting capacities at the end of the assessment period and in the intensity of parenting support they received in the 10 months following the assessment. Although we did not find any group differences on the latter, we were surprised to find that parents who received VIPP‐SD were evaluated as less capable by their interveners than parents in the RAP. Even though this could indicate that parents who received VIPP‐SD actually were less capable, the lack of other group differences (e.g., chance of recurring child maltreatment or intensity of parenting support at follow‐up) contradicts this interpretation. One explanation might be that the VIPP‐interveners were more conscious of the parenting capacities that needed to improve (i.e., aspects of parenting related to sensitivity and sensitive discipline), which may have made them more critical evaluators of these aspects than therapists who assessed families in the RAP. It should be noted here that the interveners and therapists could not be blind to families’ condition, and due to practical considerations we did not conduct an initial evaluation of parenting capacities. These aspects make it complicated to derive any strong conclusions from this finding.

The absence of beneficial effects of the VIPP‐SD protocol for PCAs in this study is unexpected, given that several researchers have argued to use attachment‐based interventions in PCAs (Cyr & Alink, 2017; Cyr et al., 2012; Lindauer et al., 2010) and two recent randomized studies have provided initial evidence that such a procedure can lead to a higher quality of placement decisions (Cyr et al., 2015; Van der Asdonk et al., 2019). An explanation for the lack of effects in the current study could be related to the quality of the RAP in the Dutch clinics. When families are referred to these clinics, they are residing there for a couple of months during which they are observed by experienced family workers and receive various treatment forms adapted to their individual needs. Families and family workers are thus highly involved in the treatment process. It is possible that within the context of this highly intensive program, VIPP‐SD does not contribute to clinically relevant improvements in PCAs, because therapists responsible for families’ placement recommendations might already be able to form a clear picture of the parenting capacities based on the regular intensive assessment procedure. The facts that therapists generally felt quite confident about their recommendations at post‐test and children's living situation at follow‐up was in most cases still consistent with the therapist's recommendation might underscore this assumption. It should be noted that this setting for PCAs is quite unique to the Netherlands and therefore the results of this study cannot be directly generalized to other countries or compared to the recent Canadian study, where the RAP was far less intensive as it included no more than 12 3‐h home visits (Cyr et al., 2015).

### 4.1 Limitations

Conducting an RCT with maltreating families in this context poses many challenges. The potential size for the study sample was limited as there were, at the time this project was conducted, only four clinics for PCAs in the Netherlands and our focus was on a specific age range. Even though we had a high response rate (79%), the sample was quite small. Another common problem with this population is a high attrition rate (e.g., Steele et al., 2019), although we still managed to reach almost two‐thirds of the families for follow‐up. Even though we used multilevel imputation to maximize power, this procedure takes the uncertainty of missing data into account by producing larger standard errors and more strict significance tests (Van Ginkel, Linting, Rippe, & van der Voort, 2019). Thus, in designing future studies it will be important to account for these issues.

A second limitation is related to the measurement of therapists’ recommendations: during data inspection we noted systematic differences in the way the initial recommendation forms were filled out by the therapists. For two therapists, 73% and 89% of the initial recommendations favored an out‐of‐home placement, whereas for the other two therapists 75% and 90% of the initial recommendations favored that the child could stay with his/her parent(s). In practice, therapists do not have to provide a recommendation regarding child placement at the start of a PCA; we solely added this measure for research purposes. Therefore, it could be that these differences were related to therapists’ interpretation of the initial recommendation form.

Third, we relied on parent reports for follow‐up data. One potential problem is that the parents who were traceable for and open to a follow‐up assessment were a selected group. Although they did not differ from parents who dropped out on demographic or target variables, it could be that after the assessment, dropped out families experienced more problems than the families who continued to participate. For instance, the majority of children (88%) were living with their parents at follow‐up; it could be that there had been more out‐of‐home placements for dyads who dropped out and that this biased the results. Another drawback of the use of parent reports is related to the validity of such reports. Previous studies have shown that abusive parents or parents with psychopathology tend to overreport their children's problem behavior (Najman et al., 2001; Reid, Kavanagh, & Baldwin, 1987), which suggests that they are not always reliable reporters of their children's actual behavior. It could be that the results of this study would have been different if we had obtained additional access to more objective reports of children's well‐being.

Finally, it is important to note that the PCA form we used in this study was restricted to assessing improvements in the parent‐child relationship and parents’ general openness toward treatment. We had constructed this assessment form to target the most important goals of VIPP‐SD and asked therapists to integrate this assessment form within their standard evaluation at the end of families’ trajectory at the clinics. In this evaluation, all relevant information is integrated, including parents’ responses to other interventions that had been provided to the family (e.g., parents’ response to trauma intervention or emotion regulation training) and risk and protective factors that are present in the family. Because the different clinics do not rely on a standardized procedure for this evaluation, we were not able to make a structural overview of how other factors in the family system (e.g., parents’ romantic relationship, social network, or intellectual abilities) contributed to therapists’ advice for the family.

### 4.2 Future directions

Even though we found no credible evidence to suggest that the PCAs incorporating the VIPP‐SD protocol outperformed the PCAs as usual, the a priori expected effect size for this study might have been too optimistic and diminished our power to detect smaller effects that could still be considered as clinically relevant. Yet, the current study may provide important reference points for future research in this area. First, by conducting this study we showed that it is possible to empirically evaluate the effectiveness of a PCA protocol in improving the quality of subsequent placement decisions through a randomized research design–which, to our knowledge, has not been done previously besides by the parallel Canadian study (Cyr et al., 2015). As we have argued previously, in future studies it will be important to carefully consider what the smallest effect size of interest is that would indicate clinically relevant results, and setting the required sample size accordingly.

A second implication is related to the unique child protection setting in which the current study was conducted: because referral to an assessment in one of the Dutch clinics is usually considered as parents’ last chance after a long trajectory of home‐based support and due to the high costs not all families can be referred there, it would be interesting to explore the effects of implementing VIPP‐SD or a similar intervention in an earlier stage. For instance, if a family is put under supervision for suspected or substantiated child maltreatment and home‐based support is imposed on the family, the VIPP‐SD assessment protocol might contribute to a better‐informed indication of their parenting capacities and therefore lead to better decisions regarding child placement. Based on two recent studies which provided initial evidence in favor of the use of attachment‐based assessments protocols (Cyr et al., 2015; Van der Asdonk et al., 2019), it would be worthwhile to further investigate the effectiveness of different implementations of this approach.

## CONFLICT OF INTEREST

The authors confirm that there are no conflict of interest associated with this publication.

## Supporting information

Supporting Information
