# Supplementary material for: Effectiveness of an attachment‐based intervention for the assessment of parenting capacities in maltreating families: A randomized controlled trial
Source: Infant Ment Health J. 2020 Jun 24;41(6):821–35. doi: 10.1002/imhj.21874 (PMC7754366; doi:10.1002/imhj.21874)
Supplement: Supplementary file 1 — Supporting Information [file IMHJ-41-821-s001.docx]

**Appendix A**

**Detailed description of sample flow throughout randomized trial**

In total, 56 families were included in the project. Following randomization, nine families dropped out (seven in the intervention group and two in the regular assessment group) for different reasons: the parent did not want to receive VIPP (*n* = 2), the child was placed in foster care before the final evaluation took place (*n* = 6), and for one mother-child dyad VIPP was stopped after one session due to individual circumstances (this dyad still took part in the post-test). The post-test did not take place for an additional seven parent-child dyads (four in the intervention group), because the parent did not want to participate anymore (*n* = 2), the child was already placed into foster care (*n* = 2), or because the family left the clinic early with a positive evaluation and could not be reached anymore (*n* = 3). The follow-up did not take place for 22 families (12 in the intervention group), because the parent did not want to participate anymore (*n* = 12), the parent was untraceable (*n* = 6), or because the parent was unavailable for an appointment (e.g., because of severe psychiatric problems) (*n* = 4).

**Appendix B**

**Multiple imputation procedures**

Four methods were used in conjunction: the ‘MI’ function in the *Amelia* package (Honaker, King, & Blackwell, 2011), the ‘mice’ function from the *mice* package (Van Buuren & Groothuis-Oudshoorn, 2011), and the ‘panImpute’ and ‘jomoImpute’ functions from the *mitml* package (Grund, Robitzsch, & Lüdtke, 2016) to assess robustness of the imputed datasets as well as access the full range of analysis options. The maximum number of iterations was set at 10 and a fixed starting seed was set for reproducibility. Pooling of results on 50 imputation sets was performed using the summary functions from *mitml* and *miceadds*, as well as using the ‘summary’ and ‘modelRandEffStats’ functions from the *merTools* package (Knowles, Frederick, & Whitworth, 2018). All models were random-intercept models; as the already small sample showed high incompleteness, random slopes were not estimated in order to avoid consecutive estimations and uncertainty under weakened model identifiability.

**References**

Grund, S., Robitzsch, A., & Lüdtke, O. (2016). Mitml: Tools for Multiple Imputation in Multilevel Modeling. In.

Honaker, J., King, G., & Blackwell, M. (2011). Amelia II: A program for missing data. *Journal of Statistical Software, 45*(7), 1-47.

Knowles, J. E., Frederick, C., & Whitworth, A. (2018). merTools: Tools for Analyzing Mixed Effect Regression Models.

Van Buuren, S., & Groothuis-Oudshoorn, K. (2011). Mice: Multivariate imputation by chained equations in R. *Journal of Statistical Software, 45*(3), 1-67.
